# The Impact of Gastroesophageal Reflux Disease and Proton Pump Inhibitor Use on the Risk of Repeat Catheter Ablation for Atrial Fibrillation

**DOI:** 10.14309/ctg.0000000000000717

**Published:** 2024-05-16

**Authors:** Jennifer X. Cai, Miguel Algara, Wai-Kit Lo, Sunil Kapur, Walter W. Chan

**Affiliations:** 1 Division of Gastroenterology, Hepatology and Endoscopy, Brigham and Women's Hospital, Boston, Massachusetts, USA;; 2 Department of Medicine, Brigham and Women's Hospital, Boston, Massachusetts, USA;; 3 Harvard Medical School, Boston, Massachusetts, USA;; 4 Division of Cardiovascular Medicine, Brigham and Women's Hospital, Boston, Massachusetts, USA.

**Keywords:** gastroesophageal reflux, GERD, atrial fibrillation, radiofrequency ablation, arrhythmia

## Abstract

**INTRODUCTION:**

Gastroesophageal reflux disease (GERD) has been associated with increased incidence/recurrence of atrial fibrillation (AF). However, the impact of GERD and proton pump inhibitor (PPI) therapy on outcomes of AF catheter ablation remains unclear. We aimed to assess the association between the presence of GERD and risk of repeat AF ablation, stratified by PPI therapy.

**METHODS:**

A retrospective cohort study was conducted on patients with paroxysmal/persistent AF undergoing initial ablation in January 2011–September 2015. GERD was defined by endoscopic findings, objective reflux testing, or clinical symptoms. The association between GERD/PPI use and time to repeat ablation was evaluated by time-to-event analysis with censoring at the last clinic follow-up within 1 year.

**RESULTS:**

Three hundred eighty-one subjects were included. Patients with GERD (n = 80) had a higher 1-year repeat ablation rate compared with those with no GERD (25% vs 11.3%, *P* = 0.0034). Stratifying by PPI use, patients with untreated GERD (37.5%) more likely needed repeat ablation compared with reflux-free (11.3%, *P* = 0.0003) and treated GERD (16.7%, *P* = 0.035) subjects. On multivariable Cox regression analyses, GERD was an independent risk factor of repeat ablation (hazard ratio [HR] 3.30, confidence interval [CI] 1.79–6.08, *P* = 0.0001). Specifically, untreated GERD was associated with earlier repeat ablation compared with no GERD (HR 4.02, CI 1.62–12.05, *P* = 0.0013). However, no significant difference in repeat ablation risk was noted between reflux-free and PPI-treated GERD groups.

**DISCUSSION:**

GERD was an independent predictor for risk of repeat AF ablation within 1 year, even after controlling for major cardiovascular comorbidities and confounders. PPI therapy modulated this risk, as repeat ablation-free survival for PPI-treated GERD was noninferior to reflux-free patients.

## INTRODUCTION

Gastroesophageal reflux disease (GERD) is one of the most common outpatient gastrointestinal diagnoses with a prevalence of 10%–15% in Western countries (1,2). The 2006 Montreal consensus defined GERD as a condition that develops when reflux of stomach contents causes troublesome symptoms and/or complications (3). GERD is further subclassified into esophageal and extraesophageal syndromes. Of the extraesophageal manifestations of GERD, airway syndromes have been the most studied to date, including GERD's impact on chronic cough, pulmonary fibrosis, lung transplantation, asthma, and chronic obstructive pulmonary disease. Evidence on other potential extraesophageal manifestations such as cardiovascular syndromes have been much less robust and remain controversial. Most frequently, theories of vagally mediated stimulation by distal esophageal refluxate and local atrial inflammation induced by anatomic proximity to reflux esophagitis have been implicated in the interaction between GERD and cardiac arrhythmias, especially atrial fibrillation (AF) (4).

As the most common arrhythmia in clinical practice, AF causes significant morbidity and mortality related to heart failure, stroke, and impaired quality of life (5). AF is often associated with structural and/or electrophysiological abnormalities; however, in 10%–15% of cases, AF presents in the absence of obvious cardiovascular comorbidities, suggesting the involvement of other potential triggers including GERD. AF has been shown in some cases to have vagally mediated etiologies, suggesting an overlap with GERD pathology. Previous cross-sectional studies have suggested a higher incidence of AF among patients with a diagnosis of GERD (6–8). Some studies have also demonstrated a higher incidence of pathologic reflux following left atrial ablation treatment of AF, citing vagal nerve damage via conductive heating (independent of direct mechanical esophageal injury) as a possible mechanism to bolster the pathophysiological link between GERD and AF (9,10). More recently, a small prospective study showed that patients with GERD who underwent left atrial ablation for paroxysmal AF may have higher risk of recurrence than those with without (11).

Thus, while these earlier studies suggest that patients with GERD not only have a higher incidence of AF but also are more likely to develop AF recurrence, the impact of GERD on objective clinical end points of AF, such as the need of repeat cardiac intervention, remains unknown. We hypothesized that GERD may be a significant risk factor for the need of repeat treatment after radiofrequency ablation for AF and that treatment of GERD may modulate this risk. The aim of this study was to assess the association between the presence of GERD and risk of short-term repeat radiofrequency ablation in patients with AF and the impact of proton pump inhibitor (PPI) therapy in modifying this risk.

## METHODS

### Study population and data collection

This was a retrospective cohort study of consecutive patients with paroxysmal and persistent AF who underwent an initial radiofrequency catheter ablation procedure at a single tertiary care center from January 2011 to September 2015. The study was approved by the Mass General Brigham Healthcare Institutional Review Board (2017P001748) before inception.

Electronic medical records were reviewed, and data were collected on the following: (i) patient demographics; (ii) reported history of cardiac comorbidities including hypertension, hyperlipidemia, diabetes mellitus, congestive heart failure, coronary artery disease, valvular disease, obstructive sleep apnea, and thyroid disease; (iii) number of repeat ablations during the follow-up period after initial treatment; (iv) number of days from initial to second ablation; (v) use of antiarrhythmic drugs (AADs) during the follow-up period; (vi) presence of AF on follow-up Holter monitoring; and (vii) diagnosis of GERD. Ablation procedure-specific factors were also collected, including (i) the experience of the operator, defined as years from completion of electrophysiology fellowship; (ii) trainee involvement during the procedure; (iii) type of anesthesia used; (iv) termination of AF achieved during the ablation procedure; and (v) total lesion (ablation) time in seconds.

All patients received prophylactic proton pump inhibitor (PPI) therapy following catheter ablation, most commonly omeprazole 40 mg twice daily for 12 weeks, as per standard institutional protocol to prevent ablation-related esophageal injury. Patients were then followed for 1 year after the initial catheter ablation event. All patients with GERD were then stratified into an untreated group, defined as those who discontinued PPI therapy following the standard 12-week prophylactic period after ablation, vs a treated group, defined as those who remained on PPI therapy of any regimen after 12 weeks during the follow-up period.

### Definitions

GERD was defined clinically by typical GERD symptoms of heartburn and/or regurgitation on reported history, objective reflux testing results if available (acid exposure time >4% or total reflux episodes >80), or upper endoscopy if available (Los Angeles grades B, C or D esophagitis, biopsy-proven Barrett's esophagus). AF was defined by the presence of arrhythmia on Holter or event monitoring or during interrogation of a pacemaker or implantable cardioverter defibrillator. The primary outcome of the study was the need for repeat catheter ablation within 1 year of the initial catheter ablation event.

### Statistical analyses

Descriptive statistics were used to examine the demographic and clinical characteristics of the study cohort. Fisher exact test for binary variables and Student *t* test for continuous variables were performed to assess for differences between subjects with and without GERD and, among the cohort with GERD, those treated and untreated by PPI. Multivariable analysis was performed using logistic regression to assess the risk of requiring repeat ablation within 1 year associated with GERD and PPI use. Time to repeat ablation was evaluated by Kaplan-Meier analysis, with censoring on the date of the last clinic visit within 1 year. No deaths occurred within 1 year of initial ablation to require additional censoring for mortality. Multivariable time-to-event analysis using a Cox proportional hazards model was applied to control for potential confounders, including age at initial ablation, sex, body mass index (BMI), coronary artery disease, heart failure, thyroid disease, and use of antiarrhythmic agents. All analyses were performed using SAS software (SAS Institute, Cary, NC).

## RESULTS

### Study population

**Figure 1.**
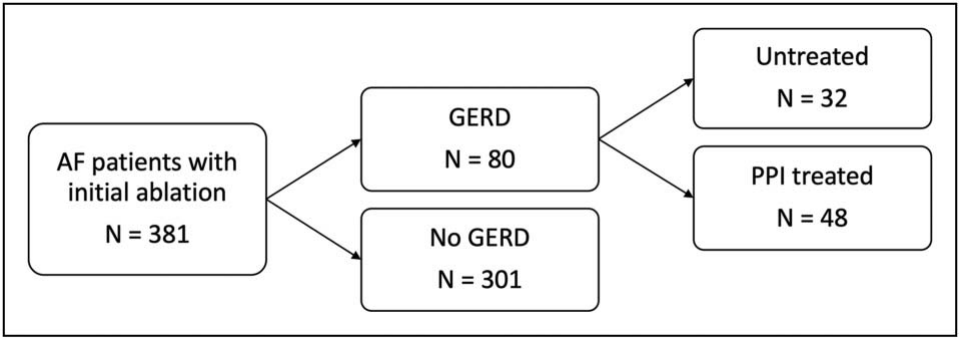
Schematic diagram of study population. Untreated patients discontinued PPI use after initial 12-week prophylaxis to prevent postablation esophageal injury while PPI-treated patients continued PPI in some form. AF, atrial fibrillation; GERD, gastroesophageal reflux disease; PPI, proton pump inhibitor.

The study cohort consisted of 381 subjects, with 263 male patients (69%) and a mean age of 61.2 years (range 20–89 years). A total of 54 subjects (14.2%) met the repeat ablation end point within 1 year. Overall, 80 subjects (21%) had a history of GERD, with 21 meeting criteria by endoscopic findings, 2 by positive reflux monitoring, and 57 by symptom history, while 301 patients (79%) were found to have no indication of GERD. Among those with GERD, 32 (40%) continued some form of PPI therapy following the standard prophylactic 12-week protocol, whereas 48 (60%) did not. No patient underwent antireflux surgery during the follow-up period. Figure 1 shows the breakdown of the study population.

A comparison of baseline characteristics revealed similar demographics; need for AADs after initial catheter ablation; and prevalence of multiple risk factors, including coronary artery disease, congestive heart failure, valvular disease, and thyroid disease, between patients with and without GERD (Table 1). Patients with GERD had a higher mean BMI compared with those without GERD (31.7 ± 6.2 vs 29.5 ± 5.8, *P* = 0.02). Among patients with GERD, no significant differences in these same baseline characteristics were detected between patients who were untreated vs treated with PPI following the initial 12-week postablation protocol.

**Table 1.** Comparison of demographic factors and clinical covariates between patients with and without GERD and treated and untreated with PPI

Baseline characteristics	GERD (n = 80)	No GERD (n = 301)	*P* value	GERD/untreated (n = 32)	GERD/treated (N = 48)	*P* value
Mean age (SD) (yr)	62.1 (±8.7)	61 (±10.1)	0.36	60.5 (8.4)	63.2 (8.8)	0.17
Sex (% male)	50 (62.5)	213 (70.8)	0.16	20 (62.5)	30 (62.5)	1.00
Thyroid disease (%)	14 (17.5)	42 (13.9)	0.55	6 (18.4)	8 (16.7)	0.47
Coronary disease (%)	26 (32.5)	89 (29.6)	0.61	7 (21.9)	19 (39.6)	0.10
Heart failure (%)	14 (17.5)	63 (20.9)	0.50	4 (12.5)	10 (20.8)	0.34
Need for AAD (%)	43 (53.0)	171 (57.0)	0.60	19 (59.4)	24 (50)	0.41
Mean BMI (SD) (kg/m^2^)	31.7 (±6.2)	29.5 (±5.8)	0.02*	31.1 (5.2)	32.1 (6.8)	0.60
Valvular disease (%)	9 (11.3)	33 (11.0)	0.94	3 (9.4)	6 (12.5)	0.66
Procedure-related factors						
Mean operator experience levels (SD) (yr)	3.15 (±0.53)	3.10 (±0.47)	0.41	2.97 (±0.18)	3.28 (±0.64)	0.003*
Trainee involved (%)	71 (88.8)	272 (90.4)	0.67	28 (87.5)	43 (89.6)	0.77
General anesthesia (%)	76 (96.2)	265 (88.0)	0.07	30 (93.8)	47 (97.9)	0.70
AF termination during ablation (%)	31 (38.8)	109 (36.2)	0.68	16 (50.0)	15 (31.3)	0.09
Total lesion (ablation) time (SD) (s)	3,326.4 (1821.1)	3,431.4 (1752.6)	0.65	3,516.6 (1,648.7)	3,214.7 (1923.8)	0.50

Patients with GERD had a higher mean BMI compared with patients without GERD. Among patients with GERD, there were no significant differences in baseline characteristics between the untreated and treated groups.

AAD, antiarrhythmic drug; AF, atrial fibrillation; BMI, body mass index; GERD, gastroesophageal reflux disease.

*P < 0.05.

There were 8 operators who performed the ablation, with similar distributions between groups. Overall, there were no significant differences in mean operator experience level, trainee involvement, procedural anesthesia administered, termination of AF during ablation, and total lesion (ablation) time between GERD and no GERD groups (Table 1). Among those with GERD, the mean operator experience level (i.e., number of years after terminal training) was slightly lower for the ablation procedures of the untreated group compared with the PPI group (2.97 ± 0.18 years vs 3.28 ± 0.64 years, *P* = 0.003). Otherwise, there were no other significant differences in operator or procedural factors between the 2 treatment groups.

### GERD vs no GERD

**Figure 2.**
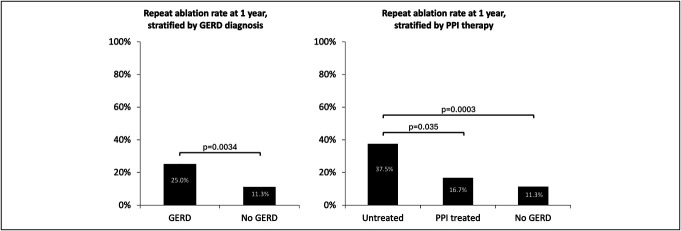
Univariate analysis of the 1-year repeat ablation rate when stratified by GERD diagnosis and PPI therapy. Patients with GERD had a significantly higher 1-year repeat ablation rate compared with patients without GERD. Patients with untreated GERD had a higher 1-year repeat ablation rate compared with both the treated GERD and no GERD groups. GERD, gastroesophageal reflux disease; PPI, proton pump inhibitor.

**Figure 3.**
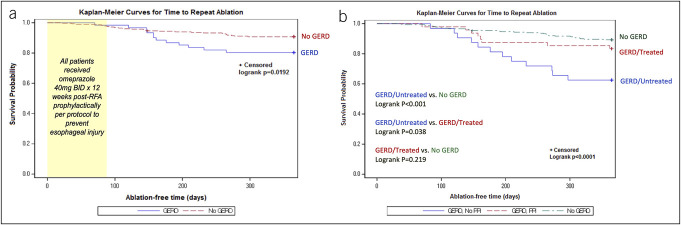
(**a**) Kaplan-Meier analysis of time to repeat ablation by GERD diagnosis in patients with atrial fibrillation who underwent initial catheter ablation. Patients with GERD were associated with decreased time to repeat ablation within 1 year compared with patients without GERD. (**b**) Kaplan-Meier analysis of time to repeat ablation within 1 year stratified by PPI therapy. Patients with untreated GERD had significantly decreased time to repeat ablation compared with treated GERD and reflux-free patients; however, there were no differences in time to repeat ablation between the treated GERD and no GERD groups. BID, twice per day; GERD, gastroesophageal reflux disease; PPI, proton pump inhibitor; RFA, radiofrequency ablation.

On univariate analysis, patients with GERD had a significantly higher 1-year repeat ablation rate compared with those without GERD (25% vs 11.3%, *P* = 0.0034) (Figure 2a). Kaplan-Meier analysis demonstrated that patients with GERD were associated with decreased time to repeat ablation within 1 year compared with patients without GERD (log-rank *P* = 0.02) (Figure 3a).

On multivariable logistic regression analysis, GERD remained an independent risk factor of repeat ablation within 1 year (odds ratio [OR] 2.57, confidence interval [CI] 1.19–5.54, *P* = 0.02), even after controlling for possible confounders, including BMI (Table 2). Time-to-event analysis with Cox regression with adjustment for potential confounders demonstrated a similar relationship, with decreased time to repeat ablation among patients with GERD compared with those without (hazard ratio [HR] 3.30, CI 1.79–6.08, *P* = 0.0001) (Table 3).

**Table 2.** Multivariable logistic regression analysis of repeat ablation within 1 year among patients with atrial fibrillation

Parameter	2a. Logistic regression	*P* value	2b. Logistic regression	*P* value
GERD	OR 2.57 (95% CI 1.19–5.54)	0.02*		
Untreated vs No GERD			OR 4.42 (95% CI 1.62–12.05)	0.02*
Treated vs No GERD			OR 1.57 (95% CI 0.55–4.46)	0.60
Age at initial ablation	OR 0.99 (95% CI 0.95–1.02)	0.41	OR 0.99 (95% CI 0.95–1.03)	0.54
Male sex	OR 1.14 (95% CI 0.51–2.58)	0.74	OR 1.14 (95% CI 0.51–2.59)	0.75
Thyroid disease	OR 1.80 (95% CI 0.88–3.67)	0.11	OR 1.71 (95% CI 0.83–3.51)	0.15
Coronary disease	OR 0.99 (95% CI 0.37–2.66)	0.98	OR 1.02 (95% CI 0.38–2.74)	0.97
Heart failure	OR 0.67 (95% CI 0.28–1.65)	0.39	OR 0.69 (95% CI 0.28–1.69)	0.41
Need for AAD after initial ablation	OR 1.30 (95% CI 0.62–2.70)	0.49	OR 1.27 (95% CI 0.61–2.67)	0.52
BMI	OR 1.03 (95% CI 0.98–1.09)	0.28	OR 1.04 (95% CI 0.98–1.10)	0.23
Valvular disease	OR 1.98 (95% CI 0.75–5.24)	0.17	OR 2.11 (95% CI 0.80–5.60)	0.13
Operator experience	OR 1.24 (95% CI 0.67–2.29)	0.49	OR 1.40 (95% CI 0.75–2.62)	0.30
Trainee involvement in ablation	OR 0.93 (95% CI 0.31–2.83)	0.90	OR 1.01 (95% CI 0.33–3.11)	0.98

The presence of GERD is predictive of risk of repeat ablation within 1 year (a). When stratified by PPI therapy (b), after controlling for demographics and comorbid conditions, patients with untreated GERD remained at higher risk of needing repeat ablation compared with those without GERD; however, patients with treated GERD did not differ from reflux-free patients in their need for repeat ablation within 1 year.

AAD, antiarrhythmic drug; BMI, body mass index; CI, confidence interval; GERD, gastroesophageal reflux disease; OR, odds ratio.

*P < 0.05.

**Table 3.** Multivariable Cox regression analysis of repeat ablation within 1 year among patients with atrial fibrillation

Parameter	3a. Cox regression	*P* value	3b. Cox regression	*P* value
GERD	HR 3.30 (95% CI 1.79–6.08)	0.0001*		
Untreated vs no GERD			HR 4.02 (95% CI 1.72–9.37)	0.0013*
Treated vs no GERD			HR 1.68 (95% CI 0.63–4.46)	0.30
Age at initial ablation	HR 0.99 (95% CI 0.96–1.03)	0.69	HR 1.00 (95% CI 0.96–1.03)	0.79
Male sex	HR 1.35 (95% CI 0.67–2.74)	0.40	HR 1.11 (95% CI 0.52–2.35)	0.79
Thyroid disease	HR 1.80 (95% CI 0.97–3.33)	0.06	HR 1.65 (95% CI 0.90–3.03)	0.11
Coronary disease	HR 0.74 (95% CI 0.29–1.84)	0.51	HR 1.01 (95% CI 0.42–2.43)	0.99
Heart failure	HR 0.45 (95% CI 0.17–1.20)	0.11	HR 0.69 (95% CI 0.31–1.57)	0.38
Need for AAD after initial ablation	HR 1.08 (95% CI 0.58–1.99)	0.81	HR 1.26 (95% CI 0.64–2.48)	0.51
BMI	HR 1.21 (95% CI 0.62–2.33)	0.58	HR 1.04 (95% CI 0.99–1.10)	0.15
Valvular disease	HR 2.07 (95% CI 0.94–4.59)	0.07	HR 2.16 (95% CI 0.91–5.16)	0.08
Operator experience	HR 1.04 (95% CI 0.58–1.86)	0.89	HR 1.30 (95% CI 0.73–2.31)	0.38
Trainee involvement in ablation	HR 0.58 (95% CI 0.23–1.44)	0.24	HR 0.86 (95% CI 0.31–2.37)	0.76

The presence of GERD is predictive of earlier repeat ablation within 1 year (a). When stratified by PPI therapy (b), patients with untreated GERD experienced earlier repeat ablation compared to those without GERD; however, patients with treated GERD did not differ from reflux-free patients in time to repeat ablation.

AAD, antiarrhythmic drug; BMI, body mass index; CI, confidence interval; GERD, gastroesophageal reflux disease; HR, hazard ratio.

*P < 0.05.

### Untreated GERD vs PPI-Treated GERD vs No GERD

When patients with GERD were stratified by use of PPI therapy, patients with untreated GERD were more likely to require repeat ablation within 1 year compared with reflux-free (37.5% vs 11.3%, *P* = 0.0003) and PPI-treated (37.5% vs 16.7%, *P* = 0.035) subjects with GERD. There was no significant difference in the need of repeat ablation at 1 year between the treated and reflux-free groups (16.7% vs 11.3%, *P* = 0.29) (Figure 2b). Time-to-event Kaplan-Meier analysis also revealed that patients with untreated GERD had a significantly shorter time to repeat ablation within 1 year compared with patients without GERD (log-rank *P* < 0.001) and those with treated GERD (log-rank *P* = 0.04) (Figure 3b). Importantly, there was no difference in the time to repeat ablation between the treated GERD and no GERD groups, with 50% subjects who reached the end point undergoing repeat ablation at 149 vs 167 days (log-rank *P* = 0.22). In addition, the separation of the Kaplan-Meier curves for risk of repeat ablation between the untreated and treated GERD groups began at around 120–130 days, which is roughly equivalent to 1 month following the standard initial 12-week prophylactic PPI regimen.

On multivariable logistic regression analysis adjusting for potential confounders, patients with untreated GERD remained at greater risk of requiring repeat ablation within 1 year compared with those with no GERD (OR 4.42, CI 1.62–12.05, *P* = 0.02); however, patients with treated GERD showed no difference in the need of repeat ablation compared with those with no GERD (OR 1.57, CI 0.55–4.46, *P* = 0.60) (Table 2). These same relationships persisted on multivariable time-to-event Cox regression analysis, with untreated GERD remaining an independent risk factor of earlier repeat ablation compared with patients with no GERD (HR 4.02, CI 1.72–9.37, *P* = 0.0013), whereas there was no difference in time to repeat ablation between the treated GERD and no GERD groups (HR 1.68, CI 0.63–4.46, *P* = 0.60) (Table 3).

### Subgroup analyses: objective evidence of GERD

We performed subgroup analyses comparing patients with objective evidence of GERD (on endoscopy or objective reflux monitoring) with those without. Increased 1-year repeat ablation rate (26.1% vs 13.4%, *P* = 0.09) and time to repeat ablation (log-rank *P* = 0.02) (see Supplementary Figure 1, http://links.lww.com/CTG/B125) were noted among patients with objective evidence of GERD, although statistical significance was not reached with the former, likely due to sample size.

When patients with objective evidence of GERD were stratified by PPI therapy, those with untreated GERD were again more likely to require repeat ablation in 1 year compared with those without objective GERD (55.6% vs 13.4%, *P* = 0.0004) and PPI-treated objective GERD (55.6% vs 7.1%, *P* = 0.01). There was again no significant difference between the treated and objective GERD-free groups (7.1% vs 13.4%, *P* = 0.50). On time-to-event analysis, patients with untreated objective GERD had significantly shorter time to repeat ablation within 1 year compared with those without objective GERD (log-rank *P* < 0.0001) and those with treated objective GERD (log-rank *P* = 0.01). No difference in time to repeat ablation was noted between the treated objective GERD and no objective GERD groups (log-rank *P* = 0.54) (see Supplementary Figure 2, http://links.lww.com/CTG/B125).

Owing to the small sample size of patients with objective evidence of GERD, multivariable subgroup analyses were not performed to avoid model overfitting.

## DISCUSSION

While many airway syndromes have been established as extraesophageal manifestations of GERD, the notion that cardiovascular syndromes such as AF may also be related to reflux continues to be debated (12). A recent comprehensive review of the evidence yielded only 8 studies in the primary literature that examined the interaction between GERD and AF, many of which were cross-sectional in nature and used self-reported questionnaires, International Classification of Diseases, 9th edition (*ICD-9*) codes, or clinical symptoms to diagnose both GERD and AF (4). In this study, we longitudinally assessed the correlation between GERD and clinical outcomes in AF and are the first to show that GERD is an independent predictor for short-term risk of repeat treatment after radiofrequency ablation for AF. Moreover, we found that PPI therapy for GERD may modulate this risk.

One of the first studies to examine a correlation between GERD and AF examined 188 outpatients with reflux, defined using a quantitative frequency scale, and found that total frequency scores were significantly higher in patients with AF compared with those without AF (6). Multivariable analysis also revealed that AF was an independent risk factor of GERD symptoms. Several early observational case series also noted a possible correlation between PPI use in patients with GERD and AF symptom reduction (13,14). The largest study thus far evaluated over 160,000 ambulatory patients from a military healthcare system and found that the prevalence of AF was higher among patients with GERD by nearly 40% (RR 1.39, 95% CI 1.33–1.45) (7). This relationship persisted after adjusting for demographic factors and common cardiovascular risk factors and conditions associated with AF. In 2012, a Taiwanese population-based, matched case-control study of nearly 30,000 patients diagnosed with GERD by upper endoscopy or 24-hour pH study revealed that AF was significantly more common among patients with GERD than those without GERD (8). More recently in 2015, a prospective study of 88 patients with paroxysmal AF who underwent left atrial ablation showed that GERD was independently associated with AF recurrence (OR 8.5, 95% CI 1.64–44.15, *P* = 0.011) (11). Notably, in this study, AF recurrence was defined primarily by patient-reported clinical symptoms, with only intermittent ambulatory cardiac monitoring.

In our study, we longitudinally followed patients with AF who underwent radiofrequency ablation and used an objective end point of repeat ablation within 1 year. Most of the cardiovascular comorbidities were similarly distributed between subjects with and without GERD in our cohort, although a higher mean BMI was noted among patients with GERD. This was not surprising as previous observational and cross-sectional studies found that increasing BMI is associated with higher frequency of GERD symptoms, with increased intragastric pressure and esophagogastric pressure gradient (15,16). Similarly, individuals with obesity are more likely to develop AF, a process thought to be mediated by an increase in left atrium size due to increased intra-atrial pressures (17,18). Given this potential for confounding, our subsequent regression modeling adjusted for BMI and other covariates that may be linked to both GERD and AF, confirming the independent association between GERD and repeat ablation. Importantly, among patients with GERD, the risk of repeat ablation was significantly lower among those who received PPI during the follow-up period compared with those who did not and was similar to those without GERD. This suggests that PPI use may modulate the increased risk of repeat ablation associated with GERD.

The Kaplan-Meier analysis demonstrated that patients with GERD had significantly decreased time to repeat ablation than patients without GERD. Notably, the 2 curves begin to diverge at approximately 120 days after ablation. At our tertiary referral center, the standard protocol among our electrophysiologists was to place all postablation patients on 12 weeks (or 90 days) of twice-daily high-dose PPI prophylactically. Hence, it is possible that the delayed divergence of the 2 curves may be in part related to some residual effects of recent PPI use, as well as treatment of reflux with use of PPI in the month following completion of the postablation prophylactic PPI regimen. Investigation of this hypothesis would be challenging as providing postablation acid suppression is the standard of care within our institution and many others, and the time interval of 12 weeks matches the blanking period, which occurs within the first 2–3 months after catheter ablation when early recurrences are common and may be heavily confounding. However, this may further support the potential impact of PPI in modulating the risk of repeat invasive cardiac intervention among AF patients with GERD and in improving their cardiac outcomes.

A few studies to date have examined the relationship between PPI use and symptomatic improvement of AF. Among the first in 2003, Weigl et al performed a retrospective study of 89 patients with upper endoscopy-confirmed reflux esophagitis who were all treated with a PPI for at least 2 months. A total of 18 patients had paroxysmal AF. Both GERD and AF-related symptoms improved in 14 patients (78%) and 5 were able to stop AAD altogether (13). In 2006, Cuomo et al conducted a case-control study of 41 patients with GERD-related symptoms, including 32 with dysrhythmias and 9 without. All patients were treated with 3 months of daily PPI therapy. In 18 (56%) of the patients with both dysrhythmia and GERD symptoms, correlation between esophageal pH and heart rate variability was observed. A significant reduction of cardiac symptoms after PPI therapy was also found in a subgroup (14). The limitations of both studies include their small sample sizes as well as the lack of a control group or one that was treated with placebo. Thus far, there have been no prospective clinical trials examining the role of PPI in AF symptom management outside of postablation prophylaxis for gastrointestinal injury.

There were limited data in the literature examining objective measures of GERD and cardiac arrhythmia. Objective testing using simultaneous 24-hour esophageal ambulatory pH and Holter monitoring was first used by Gerson et al (19) to characterize a temporal relationship between reflux and atrial arrhythmias. In that study, the authors also reported a reduction in arrhythmia symptoms following PPI therapy, although this was limited to observations in only 3 patients. A 2017 study also objectively evaluated 22 patients with paroxysmal AF using concurrent ambulatory pH and Holter monitoring (20). Episodic AF occurred in only 1 patient who did not have pathologic acid reflux based on a pH study. In addition, a total of 23 episodes of AF were documented with only a 14% correlation with reflux events. These prior studies suggest that larger prospective studies assessing the temporal relationships between GERD and AF, as well as the overall burden of both acid and non-acid reflux and arrhythmia events, are needed. Nevertheless, our study represents one of the first longitudinal studies using an objective end point to assess the relationship between GERD and AF and the potential impact of GERD treatment with PPI.

There are a few limitations to this study. First, the study included patients with AF who underwent radiofrequency ablation, likely representing a more severe phenotype of AF, thereby potentially limiting the generalizability of the data. However, these patients followed a similar ablation and postprocedure protocol, which may help minimize potential bias. In addition, the retrospective nature of the study design limited our ability to control for specific PPI formulation, dose, and frequency as well as patient adherence to acid suppression. Moreover, the definition of GERD was primarily based on clinical symptoms, with only a minority having undergone objective reflux testing or endoscopy. There is a possibility that patients with functional heartburn, visceral hypersensitivity, and dyspepsia, who often share reflux-like symptoms, may have been classified in the GERD group as a result. However, this misclassification would have likely biased the results toward the null. Our significant results despite this limitation should, thereby, strengthen our overall conclusions. In addition, defining GERD only by the presence of endoscopic evidence or positive objective reflux monitoring on subgroup analyses did not change the overall results of our findings, with increased repeat ablation among patients with objective GERD, particularly those who were untreated.

In conclusion, GERD was an independent predictor for risk of repeat catheter ablation for AF within 1 year of treatment, even after controlling for major cardiovascular risk factors and comorbidities. PPI therapy may modulate this risk, such that repeat ablation-free survival for patients with treated GERD was noninferior to reflux-free patients. These findings support a role for GERD as a potentially modifiable risk factor in AF development, recurrence, and treatment outcome. Assessment for and aggressive therapy of GERD should be considered in the routine management of recurrent and refractory AF.

## CONFLICTS OF INTEREST

**Guarantor of the article:** Walter W. Chan, MD, MPH, FACG.

**Specific author contributions:** All authors were involved in the study concept, and design and interpretation of the data. J.X.C. and M.A.: performed the primary data collection. J.X.C. and W.W.C.: authored the initial draft of the manuscript and performed statistical analysis. All authors critically revised the manuscript and approved the final copy.

**Financial support:** None to report.

**Potential competing interests:** W.W.C. served on the advisory board for Sanofi Pharmaceuticals and Regeneron Pharmaceuticals. No other authors have potential conflicts of interest to disclose.

**Study Highlights**

**WHAT IS KNOWN**

✓ Patients with gastroesophageal reflux disease (GERD) have a higher incidence of atrial fibrillation (AF) and are also more likely to develop recurrence-based AF.
✓ The impact of GERD as well as proton pump inhibitor (PPI) therapy on treatment outcomes for AF is not well elucidated, although it is commonly prescribed after AF ablation for esophageal protection.

**WHAT IS NEW HERE**

✓ GERD is an independent predictor for the objective end point of risk of repeat catheter ablation within 1 year, even after controlling for major cardiovascular comorbidities.
✓ PPI seems to modulate the risk of GERD for repeat ablation in AF: Repeat ablation-free survival for patients with treated GERD was noninferior to reflux-free patients.
✓ Our findings support a role for GERD in AF development, recurrence, and treatment outcome.

## Supplementary Material

## References

[1] Peery AF Crockett SD Barritt AS . Burden of gastrointestinal, liver, and pancreatic diseases in the United States. Gastroenterology 2015;149(7):1731–41.e3. 26327134 10.1053/j.gastro.2015.08.045 PMC4663148

[2] Vakil N. Disease definition, clinical manifestations, epidemiology and natural history of GERD. Best Pract Res Clin Gastroenterol 2010;24(6):759–64. 21126691 10.1016/j.bpg.2010.09.009

[3] Vakil N van Zanten SV Kahrilas P . The Montreal definition and classification of gastroesophageal reflux disease: A global evidence-based consensus. Am J Gastroenterol 2006;101(8):1900–43; quiz 1943. 16928254 10.1111/j.1572-0241.2006.00630.x

[4] Roman C Bruley des Varannes S Muresan L . Atrial fibrillation in patients with gastroesophageal reflux disease: A comprehensive review. World J Gastroenterol 2014;20(28):9592–9. 25071357 10.3748/wjg.v20.i28.9592 PMC4110594

[5] Hwang JJ Lee DH Yoon H . Is atrial fibrillation a risk factor for gastroesophageal reflux disease occurrence? Medicine (Baltimore) 2015;94(43):e1921. 26512618 10.1097/MD.0000000000001921 PMC4985431

[6] Shimazu H Nakaji G Fukata M . Relationship between atrial fibrillation and gastroesophageal reflux disease: A multicenter questionnaire survey. Cardiology 2011;119(4):217–23. 21985841 10.1159/000331497

[7] Kunz JS Hemann B Edwin Atwood J . Is there a link between gastroesophageal reflux disease and atrial fibrillation? Clin Cardiol 2009;32(10):584–7. 19911354 10.1002/clc.20660 PMC6653088

[8] Huang CC Chan WL Luo JC . Gastroesophageal reflux disease and atrial fibrillation: A nationwide population-based study. PLoS One 2012;7(10):e47575. 23077642 10.1371/journal.pone.0047575 PMC3471851

[9] Nolker G Ritscher G Gutleben KJ . Esophageal acid levels after pulmonary vein isolation for atrial fibrillation. Pacing Clin Electrophysiol 2009;32(Suppl 1):S228–30. 19250102 10.1111/j.1540-8159.2008.02292.x

[10] Martinek M Hassanein S Bencsik G . Acute development of gastroesophageal reflux after radiofrequency catheter ablation of atrial fibrillation. Heart Rhythm 2009;6(10):1457–62. 19716773 10.1016/j.hrthm.2009.06.022

[11] Lioni L Letsas KP Efremidis M . Gastroesophageal reflux disease is a predictor of atrial fibrillation recurrence following left atrial ablation. Int J Cardiol 2015;183:211–3. 25675903 10.1016/j.ijcard.2015.01.083

[12] Floria M Drug VL. Atrial fibrillation and gastroesophageal reflux disease: From the cardiologist perspective. World J Gastroenterol 2015;21(10):3154–6. 25780320 10.3748/wjg.v21.i10.3154 PMC4356942

[13] Weigl M Gschwantler M Gatterer E . Reflux esophagitis in the pathogenesis of paroxysmal atrial fibrillation: Results of a pilot study. South Med J 2003;96(11):1128–32. 14632362 10.1097/01.SMJ.0000084294.77504.4B

[14] Cuomo R De Giorgi F Adinolfi L . Oesophageal acid exposure and altered neurocardiac function in patients with GERD and idiopathic cardiac dysrhythmias. Aliment Pharmacol Ther 2006;24(2):361–70. 16842463 10.1111/j.1365-2036.2006.02987.x

[15] Jacobson BC Somers SC Fuchs CS . Body-mass index and symptoms of gastroesophageal reflux in women. N Engl J Med 2006;354(22):2340–8. 16738270 10.1056/NEJMoa054391 PMC2782772

[16] Pandolfino JE El-Serag HB Zhang Q . Obesity: A challenge to esophagogastric junction integrity. Gastroenterology 2006;130(3):639–49. 16530504 10.1053/j.gastro.2005.12.016

[17] Nalliah CJ Sanders P Kottkamp H . The role of obesity in atrial fibrillation. Eur Heart J 2016;37(20):1565–72. 26371114 10.1093/eurheartj/ehv486

[18] Mahajan R Lau DH Brooks AG . Electrophysiological, electroanatomical, and structural remodeling of the atria as consequences of sustained obesity. J Am Coll Cardiol 2015;66:1–11. 26139051 10.1016/j.jacc.2015.04.058

[19] Gerson LB Friday K Triadafilopoulos G. Potential relationship between gastroesophageal reflux disease and atrial arrhythmias. J Clin Gastroenterol 2006;40(9):828–32. 17016140 10.1097/01.mcg.0000225571.42890.a5

[20] Coutinho EL Herbella FAM Lovato CAV . Objective evaluation of gastroesophageal reflux disease in patients with paroxysmal atrial fibrillation. World J Surg 2018;42(5):1458–62. 29134307 10.1007/s00268-017-4337-4

