# Supplementary material for: The Impact of Gastroesophageal Reflux Disease and Proton Pump Inhibitor Use on the Risk of Repeat Catheter Ablation for Atrial Fibrillation
Source: Clin Transl Gastroenterol. 2024 May 16;15(6):e00717. doi: 10.14309/ctg.0000000000000717 (PMC11196073; doi:10.14309/ctg.0000000000000717)

Supplementary Figure 1: Kaplan-Meier analysis of time to repeat ablation by objective evidence of GERD in patients with AF who underwent initial catheter ablation. Patients with objective evidence of GERD on upper endoscopy or objective reflux monitoring tests were associated with decreased time to repeat ablation within one year compared to patients without objective GERD.

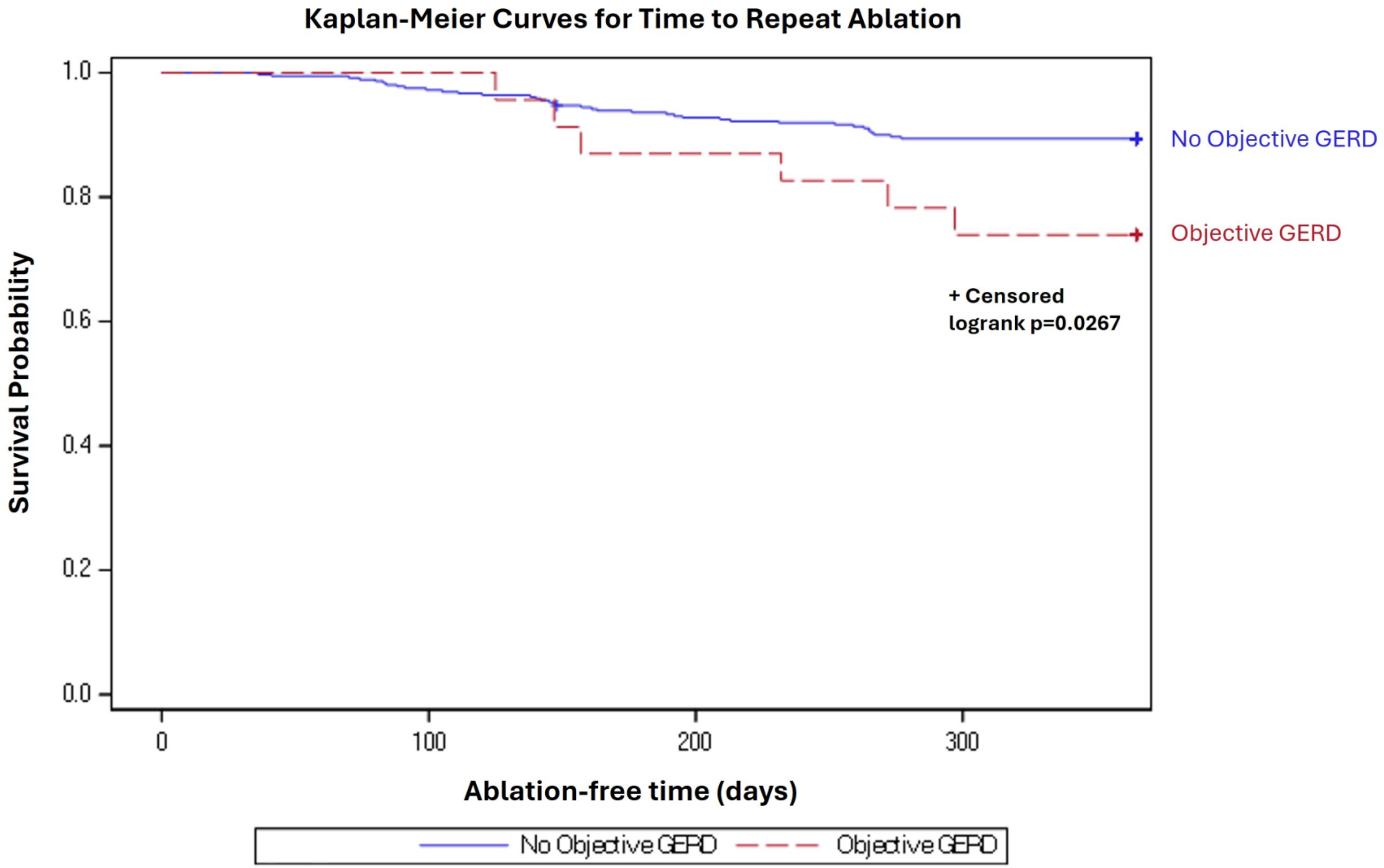

Supplementary Figure 2: Kaplan-Meier analysis of time to repeat ablation within one year stratified by PPI therapy of patients with objective evidence of GERD. Untreated objective GERD patients had significantly decreased time to repeat ablation compared to treated objective GERD and patients with no known objective evidence of GERD; however, there were no differences in time to repeat ablation between the treated objective GERD and no objective GERD groups.

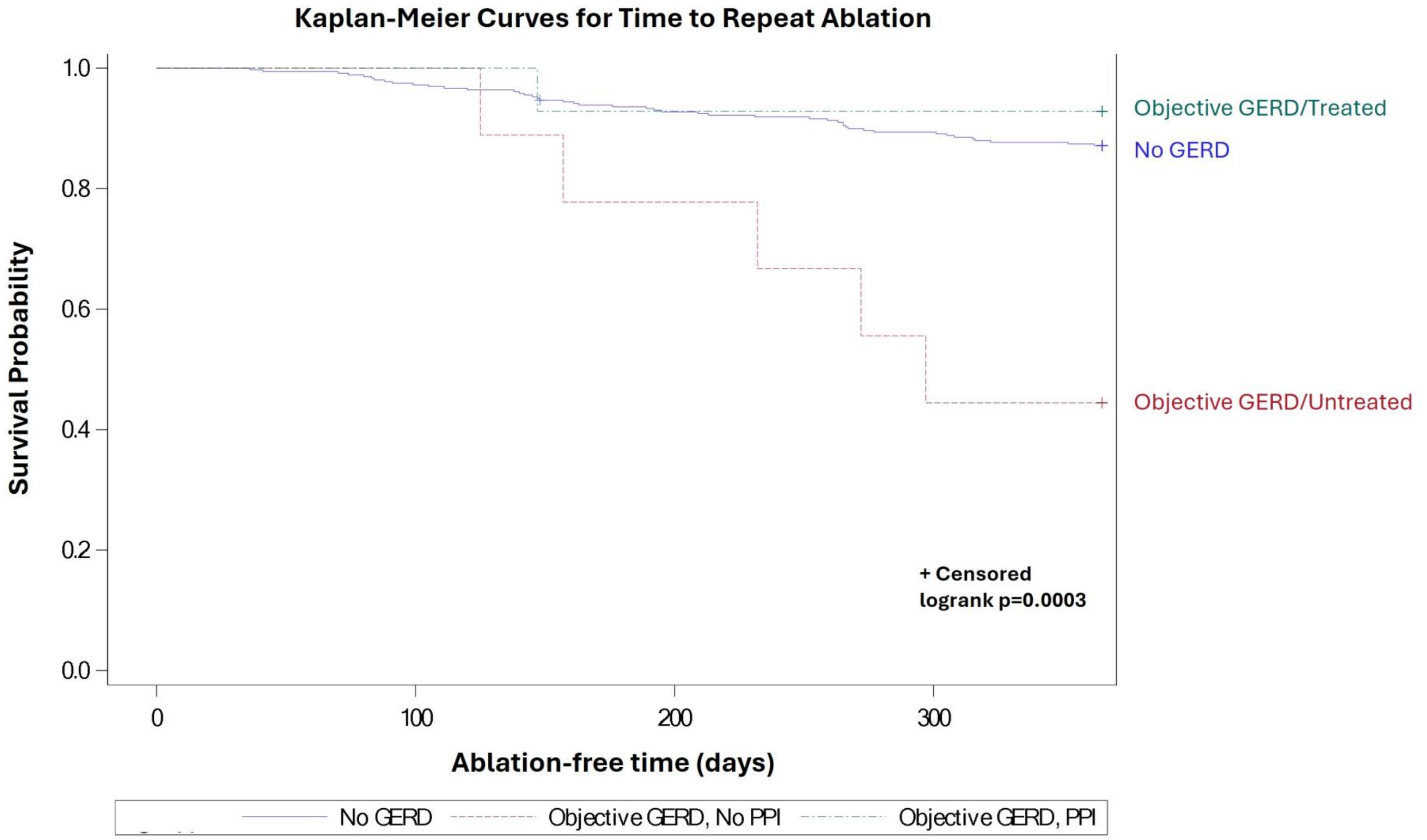

Supplement: Supplementary file 1 [file ct9-15-e00717-s001.pdf]
